# Exploring the association between secondhand smoke exposure and hearing loss among U.S. nonsmokers

**DOI:** 10.1371/journal.pone.0331105

**Published:** 2025-09-02

**Authors:** Aashish Batheja, Daniel H. Coelho

**Affiliations:** Department of Otolaryngology – Head & Neck Surgery, Virginia Commonwealth University School of Medicine, Richmond, Virginia, United States of America; Universidad de Chile, CHILE

## Abstract

**Objective:**

Secondhand smoke (SHS) exposure has been implicated as a risk factor for hearing loss. However, there is a relative paucity of inconsistent findings with limited frequency-specific details. The goal of this study is to better characterize the relationship between SHS exposure and hearing loss among adult nonsmokers in the U.S.

**Study design:**

Cross-sectional.

**Setting:**

2015-2016 NHANES cycle.

**Patients:**

1644 nonsmokers between ages 20 and 69 and without diabetes, stroke, or heart disease.

**Intervention:**

Serum cotinine level (ng/ml) indicated SHS exposure.

**Main outcome measures:**

Outcomes were hearing thresholds at low-frequencies and high-frequencies as well as hearing loss defined by hearing threshold 20 dB in the better ear. Linear regressions between hearing thresholds and SHS exposure stratified by Body Mass Index (BMI) category and controlled for socio-demographic variables. Logistic regression modeling hearing loss by SHS exposure controlled for the same.

**Results:**

SHS exposure was associated with elevated hearing thresholds at low-frequencies (β = 0.47, p = .03) only in the obese (BMI 30) population. SHS exposure was associated with greater odds of hearing loss (Odds Ratio: 1.17, 95% Confidence Interval: 1.06–1.29, p = .005) and demonstrated a dose-response relationship.

**Conclusion:**

While SHS exposure was associated with hearing loss and showed a dose-response relationship, its relationship with hearing thresholds was not demonstrated across all hearing frequencies or BMI categories. Additional research is needed to establish the clinical significance of these findings and clarify the role of obesity in this relationship.

## Introduction

Although smoking is a well-established risk factor of sensorineural hearing loss [1–3], less is known about the effects of secondhand smoke (SHS) exposure on hearing loss. Interestingly, much of what little is known about SHS and hearing may come from children – a more vulnerable population with respect to SHS exposure. Lalwani et al. reported that SHS exposure was associated with increased risk of unilateral sensorineural hearing loss at low-frequencies for adolescents aged 12–19 [4]. Another study of children between the ages of 5 and 11 discovered that high levels of SHS exposure were linked to a greater risk of hearing loss [5]. Additionally, one study of adolescents aged 12–15 asserted that prenatal smoke exposure was related to unilateral low-frequency hearing loss [6].

Much about the relationship between SHS and hearing loss in the adult population has not yet been fully clarified. Cruickshanks and colleagues reported nonsmokers with a household member who smoked had greater odds of hearing loss compared to nonsmokers who did not live with a smoker [3]. Others found speech-recognition to be impaired in non-smoking middle-aged adults who report spending greater time around SHS exposure [7].

A variety of mechanisms have been proposed regarding how smoking in general may facilitate hearing loss, including blood vessel vasospasm, arteriosclerosis, and cochlear ischemia [8]. On the other hand, current evidence indicates that SHS exposure may primarily impair auditory function through direct toxicity [9]. Indeed, both the nicotine and other chemical substances in cigarette smoke can be ototoxic [7]. Other studies of mice have shown that cigarette smoke exposure may increase oxidative stress in cochlear tissue [10]. However, similar studies in human populations are lacking and a further mechanistic understanding of SHS and hearing loss is limited.

In an attempt to more accurately assess SHS exposure, some studies have utilized serum cotinine levels as a biomarker of SHS exposure. Fabry et al. showed that cotinine level is associated with hearing loss at low- and mid- frequencies for both “never smokers” and former smokers [8]. SHS exposure was also significantly linked to high-frequency hearing loss only in former smokers [8]. A different analysis of hearing thresholds found that increased cotinine level was associated with elevated hearing thresholds at high- and low-frequencies, yet this association applied only to the obese population [11]. The connection between obesity, SHS exposure, and hearing loss, especially in the nonsmoking population, has yet to be thoroughly elucidated.

While there is evidence suggesting the association between SHS exposure and hearing loss in the adult population, to date no studies have demonstrated that this association exists for all frequencies and populations studied. Moreover, these studies are varied in their analytical techniques and criteria for hearing loss, further complicating comparability of results. Given that prevalence of SHS exposure in nonsmoking adults exceeds 20% [12] and that hearing loss can negatively affect employment, cognition, and mental health [13], there is a need to better understand the potential relationship between SHS exposure on hearing loss as well as the specific frequencies and populations that may be most affected. From a public health standpoint, the deleterious effects of smoking are well-recognized for individuals that smoke, but its potential health impact on nonsmokers is often underemphasized. Further knowledge regarding this issue may inform valuable preventative measures to support this potentially vulnerable population. The purpose of this analysis was to more thoroughly characterize the relationship between SHS exposure and hearing loss in adult nonsmokers in the U.S.

## Materials and methods

In this cross-sectional study, the National Health and Nutrition Examination Survey (NHANES) results for the 2015−2016 cycle were analyzed. NHANES is annual survey of the civilian noninstitutionalized population with “a complex, multistage, clustered design using unequal probabilities of selection.” [14] More recent survey cycles did not collect audiometric data for the target age demographic (20–69 years) or were limited in scope and generalizability to the national population due to the COVID-19 pandemic. Interviews were performed for 9971 of the 15327 eligible individuals [14]. 5204 participants were excluded due to being outside the target age range. An additional 3123 participants were excluded for missing or abnormal data in other categories, described below. Notable categories with missing data (participants may have missing data in multiple categories concurrently) included current smoking status (2842 missing values), incomplete audio exam (746 missing values), family income to poverty ratio (481 missing values), and serum cotinine level (432 missing values). Ultimately, 1644 nonsmokers between ages 20 and 69 and without other risk factors for hearing loss (e.g., diabetes, stroke, or cardiovascular disease) were included in the study. This study is considered eligible for exemption by our university's IRB under Exemption 45 CFR 46.104(d)(2).

### Study population

Participants were excluded for an incomplete audio exam, abnormal tympanometry (Type B or C as read by an audiologist), abnormal otoscopy, or presence of ear tubes. Medical conditions such as cardiovascular disease, stroke, and diabetes also precluded participants from inclusion to allow for a focus on investigating the unique connection of obesity with SHS and hearing loss in a population without the overt characteristics of “a multifactorial metabolic disorder.” [11] History of stroke and diabetes were self-reported, and cardiovascular disease was defined by self-reported history of angina, coronary artery disease, congestive heart failure, or myocardial infarction [11]. Additionally, diabetes was indicated by a Hemoglobin A1C of at least 6.5%, a fasting glucose of at least 126 mg/dL, or a 2 hour oral glucose tolerance test of at least 200 mg/dL [15].

Participants were divided into Current, Former, and Never smokers based on definitions from the Centers for Disease Control and Prevention (CDC) [16]. Current smokers were defined as participants who have smoked at least 100 cigarettes in their life and currently smoke “Every day” or “Some days.” Former smokers were defined as participants who have smoked at least 100 cigarettes in their life but do not currently smoke at all. Never smokers were defined as participants who have not smoked at least 100 cigarettes in their life.

Because current smoking status was self-reported, serum cotinine levels were used for additional verification of nonsmoking status. Cotinine is a metabolite of nicotine with a half-life of roughly 15–20 hours, making it a suitable biomarker for smoke exposure [17]. The final designation of “Nonsmoker” was assigned to Former and Never smokers with serum cotinine values less than or equal to 10 ng/ml [11,18]. Only Nonsmokers were included in the study population. Lastly, participants were excluded if they lacked data on age, gender, race, income, noise exposure, or Body Mass Index (BMI).

### SHS exposure

Self-reported questionnaires regarding SHS exposure are convenient and cost-effective, but are subject to recall and social desirability bias [19]. Indeed, self-reports may lead to underestimation of SHS exposure [20]. Thus, serum cotinine levels (ng/ml) were used as a marker of SHS exposure. Although often considered a “gold standard” for SHS exposure, important limitations of cotinine include natural variations in nicotine metabolism between individuals as well as a short half-life which may underestimate cumulative tobacco smoke exposure [21,22]. Quantities below the lower limit of detection (LLOD) of 0.015 ng/ml were replaced with the value of LLOD/sqrt(2), equivalent to 0.011 ng/ml [17]. Serum cotinine levels were natural log-transformed (ln cotinine) due to initial right skew, consistent with prior studies [8,23]. For reference, it has been estimated that an hour of SHS exposure per day corresponds to an approximate 0.45 ng/ml increase in serum cotinine levels [24].

### Hearing measures

For the 2015–2016 cycle, NHANES collected audiometric data for individuals between the ages of 20 and 69 [25]. Hearing thresholds at low- and high-frequencies were calculated for each ear. Low-frequency hearing threshold was calculated as the pure-tone average of 0.5 kHz, 1 kHz, and 2 kHz [11]. High-frequency hearing threshold was calculated as the pure-tone average of 3 kHz, 4 kHz, 6 kHz, and 8 kHz [11]. Values noted as “No response” were re-coded to the maximum threshold tested for that frequency [26].

The presence of absence of hearing loss was defined by World Health Organization (WHO) guidelines with normal hearing was defined as a hearing threshold of less than 20 dB while hearing loss was defined as a hearing threshold greater than or equal to 20 dB in the better ear [13]. Additionally, a small number of cases considered as unilateral hearing loss by the WHO (hearing threshold 35 dB in the worse ear despite no hearing loss in the better ear) were defined as hearing loss (n = 27) [13]. Most cases of hearing loss in the study population demonstrated high-frequency hearing loss, reflecting the fact that most cases of hearing loss first manifest at high frequencies and high-frequency hearing loss may be a harbinger of later low-frequency hearing loss [27]. To account for the limited number of solely low-frequency hearing loss cases, an overall measure of hearing loss at any frequency was utilized.

### Other variables

Income, which has been associated with hearing loss [28], was measured as family income to poverty ratio [29]. Noise exposure was classified as present when respondents reported ever having either job exposure to loud noise or off-work exposure to loud noise. Job exposure to loud noise was defined as loud noise for “4 or more hours a day, several days a week” while off-work exposure to loud noise was defined as “very loud noise or music for 10 or more hours a week” outside of a work setting [25]. BMI categories were defined as normal weight (18.5 to <25), overweight (25 to < 30), and obese ( 30) [11].

### Statistical considerations

Linear regression analyses between hearing thresholds and SHS exposure controlled for age, gender, race, income, and noise exposure. They were also stratified by BMI category as done by Y.-Y. Lin et al. in their study of hearing thresholds [11]. For the logistic regression, the outcome of hearing loss was derived from the World Report on Hearing as outlined in the “Hearing Measures” section [13]. The multivariable logistic regression model controlled for age, gender, race, income, and noise exposure. Limited subgroup size precluded stratification by BMI. Analyses stemming from small sample sizes may have limited validity as a result of the nature of the NHANES sampling techniques [26]. BMI was controlled for in the regression model instead. Customized odds ratios for hearing loss by cotinine level were also calculated. In order to account for the complex clustered design of NHANES, appropriate sampling weights and design variables were used [30]. SAS™ 9.04 software was used for statistical analyses.

## Results

**Table 1** describes the demographic characteristics of the study population. For NHANES 2015–2016, the Hispanic, non-Hispanic Black, and non-Hispanic Asian populations were over-sampled [14].

**Table 1:** Demographics of study participants (n = 1644).

	N (%)	Mean (SEM)
Age		42.64 (0.61)
Gender Male Female	648 (39.42%) 996 (60.58%)	
Race White Black Asian Hispanic Other	500 (30.41%) 263 (16.00%) 281 (17.09%) 323 (19.65%) 277 (16.85%)	
Income (Family income to poverty ratio)		3.33 (0.10)
Loud Noise Exposure Yes No	494 (30.05%) 1150 (69.95%)	
BMI Normal weight Overweight Obese	480 (29.20%) 530 (32.24%) 634 (38.56%)	
Cotinine level (ng/mL)		0.18 (0.02)
Low-frequency Pure-Tone Average (dB)		7.34 (0.40)
High-frequency Pure-Tone Average (dB)		16.32 (0.89)
Hearing Loss No Yes	1240 (75.43%) 404 (24.57%)	

**Table 2** shows SHS exposure is associated with elevated hearing thresholds at low-frequencies (β = 0.47, p = .03) only in the obese population. **Table 3** shows SHS exposure was associated with a small, but statistically significant greater odds of hearing loss (Odds Ratio: 1.17, 95% Confidence Interval: 1.06–1.29). Older age (OR: 1.13, 95% CI: 1.11–1.15) was another positive predictor of hearing loss. Being female (OR: 0.42, 95% CI: 0.28–0.62) or Black (OR: 0.39, 95% CI: 0.22–0.69) were negative predictors of hearing loss.

**Table 2:** Beta (β) coefficients for linear regressions for hearing thresholds at low- and high-frequencies.

	Low-frequency Hearing Thresholds (n = 1644)			High-frequency Hearing Thresholds (n = 1644)		
	Normal-weight	Overweight	Obese	Normal-weight	Overweight	Obese
Age	**0.31 (0.11, 0.50)** ^**^	**0.24 (0.13, 0.35)** ^**^	**0.22 (0.14, 0.31)** ^***^	**0.72** **(0.50, 0.94)** ^***^	**0.69** **(0.53** **, 0.85** )^***^	**0.69** **(0.54** **, 0.85)** ^***^
Female ref = Male	1.63 (−2.07, 5.32)	−0.11 (−1.54, 1.32)	−0.70 (−2.74, 1.34)	−4.18 (−9.35, 0.98)	**−6.26** **(−8.60, −3.92)** ^***^	**−6.39** **(−9.64** , −3.15)^**^
Black race ref = White	−0.59 (−2.71, 1.52)	−1.58 (−3.39, 0.23)	−1.96 (−3.50, −0.41)*	**−4.69** **(−7.53** **, −1.85** )^**^	**−5.17** (−7.42, −2.92)^**^	**−4.34** **(−7.35** , −1.32)^**^
Other race ref = White	1.25 (−0.66, 3.16)	−0.67 (−2.32, 0.98)	−0.60 (−2.22, 1.01)	**−2.17** **(−4.23** , −0.11)*	−0.33 (−2.72, 2.07)	−0.40 (−3.19 , 2.40)
Income	−0.36 (−1.43, 0.71)	−0.87 (−1.67, −0.07)*	−0.21 (−0.84, 0.42)	**−1.20** **(−2.31** , −0.09)*	−0.78 (−1.60, 0.054)	−0.16 (−0.99 , 0.66)
Loud noise exposure ref = No	2.18 (−3.80, 8.15)	0.55 (−1.03, 2.14)	0.95 (−0.79, 2.69)	0.81 (−5.63, 7.25)	1.42 (−1.93, 4.77)	0.66 (−2.48 , 3.80)
Ln cotinine	−0.01 (−0.86, 0.83)	−0.31 (−1.00, 0.38)	0.47 (0.04, 0.89)*	0.26 (−0.78 , 1.30)	−0.08 (−0.81, 0.65)	1.45 (−0.14 , 3.03)

*significant at p < .05,

**significant at p < .01,

***significant at p < .001

**Table 3:** Multivariable logistic regression for hearing loss (n = 1644).

	β coefficient	Adjusted Odds Ratio^*^ (95% CI)	p – value
**Age**	**0.12**	**1.13 (1.11 - 1.15)**	**p < .001**
Gender **Female** ref = Male	**−0.88**	**0.42 (0.28 - 0.62)**	**p < .001**
Race **Black** Other ref = White	**−0.94** −0.16	**0.39 (0.22–0.69)** 0.85 (0.54–1.35)	**p = .003** p = .47
Income	−0.06	0.94 (0.82 - 1.08)	p = .35
Loud Noise Exposure Yes ref = No	0.29	1.34 (0.90 - 2.00)	p = .14
BMI	0.02	1.02 (0.99 - 1.06)	p = .21
**ln Cotinine** ^a^	**0.15**	**1.17 (1.06 - 1.29)**	**p = .005**

*Adjusted Odds Ratio = e^B^

^a^Odds ratio and confidence interval for 1 log unit increase

**Fig 1:**
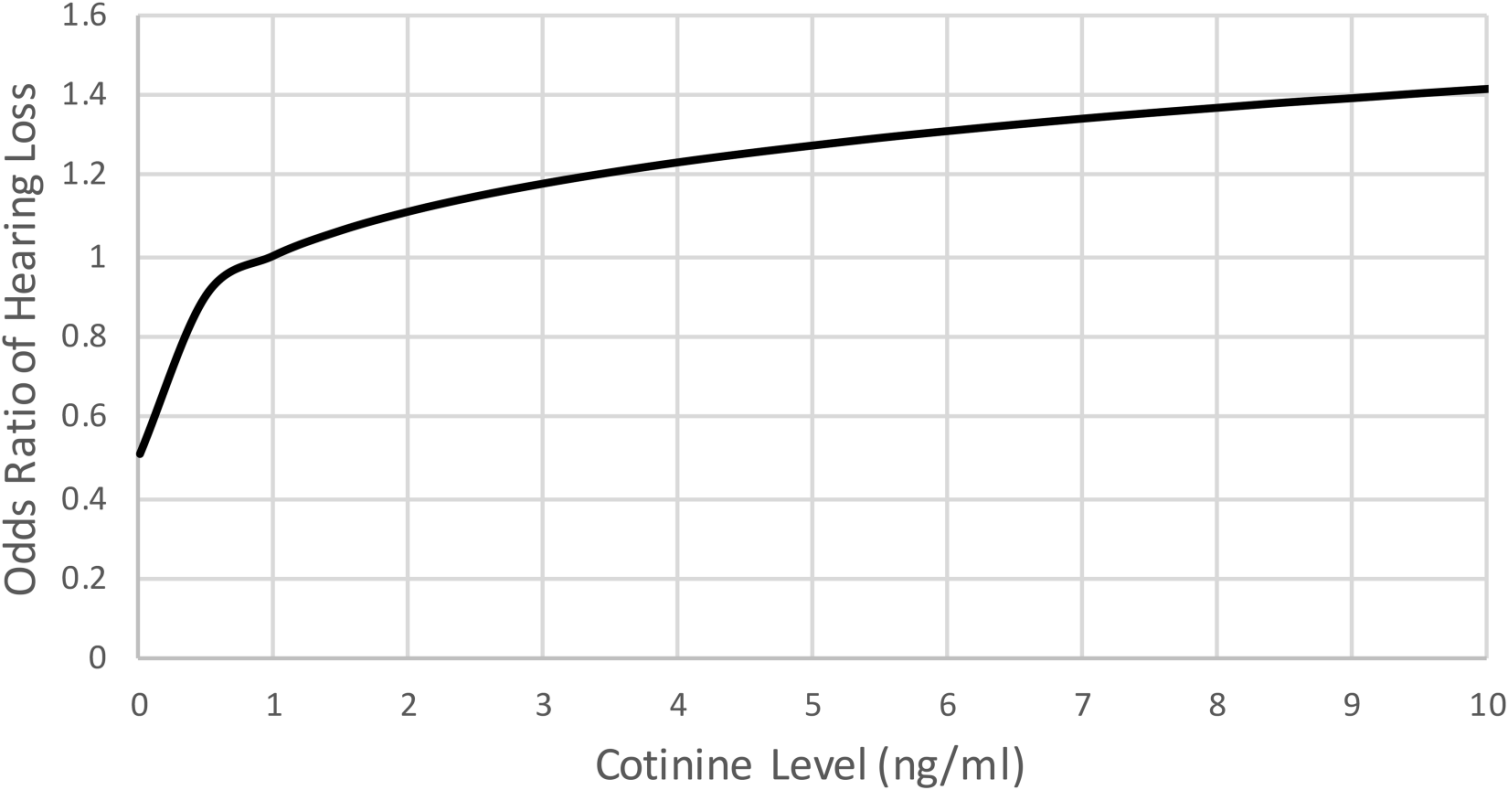
Odds Ratio for Hearing Loss by Serum Cotinine Level (ng/ml). Odds ratio for hearing loss increases as serum cotinine level increases.

**Fig 1** shows the rise in the odds ratio for hearing loss with increasing cotinine level. The odds ratio for hearing loss increased from 0.50 at a cotinine level of 0.011 ng/ml to 1.422 at a cotinine level of 10 ng/ml.

## Discussion

There are some notable differences when comparing the demographic distribution of the study population of isolated nonsmokers to the general population. 38.56% of the study population was classified as obese. This is lower than the 47.3% prevalence of obesity for adults over the age of 20 in the U.S. noted in another analysis of the NHANES 2015–2016 cycle that did not exclude individuals with diabetes, stroke, or cardiovascular comorbidities [31]. 32.24% of the study population was classified as overweight, which is similar to the 31.6% prevalence for adults over the age of 20 in the U.S. noted in that same NHANES 2015–2016 cycle analysis [31]. Most of the study population (75.43%) did not present with hearing loss. One study of U.S. adults aged 20–69 years suggested rates of hearing loss up to nearly 17%, although a threshold of 25 dB was utilized [32]. In addition to differing threshold values, variations in exclusion criteria may also contribute to the discrepancy in hearing loss rates.

In the general population, approximately 84.5% of adults were nonsmokers in 2016 [33]. Another study indicates a greater prevalence of nonsmoking amongst females as compared to males (83.8% vs. 79.4%) and amongst Hispanic individuals compared to Non-Hispanic White individuals (87% vs. 80.8%), largely driven by the greater proportion of Never smokers [34]. The present study of nonsmoking individuals demonstrates a predominance of females but differs in that the plurality of individuals self-report as White.

This analysis evaluates the association of SHS exposure on two different measures of hearing: quantitative hearing thresholds and categorical presence or absence of hearing loss. Overall, both the hearing threshold and hearing loss analyses provide support for certain associations between SHS exposure and hearing loss. These results align with previous studies demonstrating the association of SHS exposure to hearing threshold shifts as well as increased odds of hearing loss [8,11]. Importantly, our study supports the hearing threshold and hearing loss hypotheses with a common data set and statistical techniques. While Fabry et al. and Y.-Y Lin et al. both analyzed the NHANES 1999–2004 cycles, data management and statistical techniques varied between the studies [8,11]. In addition to providing a more current evaluation by using the 2015–2016 dataset and updated WHO guidelines, the present study utilizes consistent classifications and analyses across all outcomes measured. Our study also includes covariates absent in these studies, such as income.

SHS exposure was associated with elevated hearing thresholds at low-frequencies (β = 0.47, p = .03) only in the obese population. This statistically significant association corroborates one previous study noting threshold shifts only in the obese populations [11], although that study also found threshold shifts at high-frequencies. Of note, SHS exposure was positively associated with high-frequency hearing threshold but did not reach statistical significance at p = .07 in our study. Notably, our study focused on the better ear rather than the worse ear [13]. Since elevated hearing thresholds in the better ear correspond to bilateral threshold shifts, it is likely a more stringent criteria than using the worse ear that includes unilateral as well as bilateral hearing threshold shifts. It is possible that a larger subgroup could detect a statistically significant association for threshold shifts at high-frequencies.

The exact mechanisms by which obesity may modulate the relationship between SHS exposure and hearing loss have yet to be determined. It has been proposed that SHS exposure and obesity may exert a synergistic negative effect on hearing through shared biochemical pathways involving atherosclerosis, oxidative stress, and inflammation [11]. For instance, decreased blood flow in the cochlear region may damage the stria vascularis and impair cochlear function [11]. Given the relatively large magnitude and near-significance of threshold shifts at high-frequencies, it would be premature to suggest a specific biophysical pathway favoring low-frequency over high-frequency threshold shifts.

The β coefficient indicates that for each unit increase in ln cotinine level, low-frequency hearing threshold increases by 0.47 dB. Y.-Y. Lin et al. reported lower β coefficients than those in our study but did not comment on the clinical significance of their findings [11]. Comparing effect sizes between studies is further complicated by differences in analytical techniques, as Y.-Y. Lin et al. categorize serum cotinine into tertiles rather than treating it as continuous [11]. Given that a hearing threshold shift of at least 10 dB is considered “significant” hearing loss [35], this association may be statistically but not clinically significant. However, the real-world clinical significance of this association may depend on other factors like the duration of persistently elevated cotinine levels in an individual, which would require a longitudinal assessment.

SHS exposure was associated with a small, but statistically greater degree of overall hearing loss (Odds Ratio: 1.17, 95% Confidence Interval: 1.06–1.29). The magnitude of this odds ratio is comparable to the odds ratios reported by Fabry et al., which range from 1.08 to 1.40 depending on the hearing frequencies and populations (“never smokers” or former smokers) considered [8]. The results also suggest a dose-response relationship between SHS exposure and hearing loss, as evidenced in prior studies [4,7,11]. The odds ratio of hearing loss was over 1.2 at a cotinine level of 3.5 ng/ml and over 1.4 at a cotinine level of 9.5 ng/ml. The odds ratio increased most quickly between cotinine levels of 0.011 ng/ml to roughly 1 ng/ml, after which it continued to show a steady, albeit less steep, rise with increasing cotinine level. Baltar et al. reported that every hour of SHS exposure per day is associated with a 0.45 ng/ml increase in serum cotinine levels [24]. A crude estimation assuming a baseline serum cotinine level at the LLOD of 0.015 ng/ml and 5 hours of SHS exposure a day results in a serum cotinine level of 2.265 ng/ml and a corresponding odds ratio of hearing loss below 1.15 per our study. While this is reassuring for those exposed to SHS for limited periods of time, it should be noted that this calculation does not account for important factors such as proximity to SHS exposure, overall number of days of SHS exposure, and variations in baseline serum cotinine level.

Subgroup size limitations combined with the nature of NHANES data sampling precluded stratification by BMI in this logistic regression model. The potentiating nature of obesity on the association between SHS exposure and hearing threshold suggests the same influence may exist when analyzing SHS exposure and clinical hearing loss. Still, neither Fabry et al. nor our study could draw an affirmative conclusion on how much of the association between SHS exposure and clinical hearing loss is driven by a potentiating effect of obesity [8].

Younger age and being female were consistently associated with lower hearing thresholds and decreased odds of hearing loss, which is strongly supported by existing literature [36–40]. The low number of Black individuals with hearing loss relative to other races is consistent with literature showing Black race is “substantially protective against hearing loss.” [39] Indeed, Black race was a statistically significant negative predictor of hearing thresholds and hearing loss in our models.

BMI was not significantly associated with hearing loss severity in this analysis. While there is evidence for the independent association of BMI with hearing thresholds, some studies have not found a significant association [41,42]. One explanation is that Fat Mass Index (FMI), rather than BMI, may be a better indicator of metabolic dysfunction that can contribute to hearing loss [41]. Future studies should include FMI in addition to BMI as a measure. The pathologic mechanisms by which obesity may damage hearing are mainly vascular in nature [41]. One narrative review asserts that the much of the relationship between obesity and hearing loss is likely due to comorbidities of obesity like cardiovascular disease and diabetes [43]. Participants with these comorbidities were excluded from our study population, perhaps explaining the lack of significant association between BMI and hearing loss. Indeed, more research is needed on the link between obesity and hearing loss in patients without overt and detectable medical comorbidities.

Interestingly, there was no association between loud noise exposure and hearing loss despite an abundance of evidence demonstrating the existence of a strong relationship [44,45]. However, this is consistent with previous studies with the NHANES dataset that also lack this association for most populations assessed [8,26]. A likely explanation is that loud noise exposure was self-reported and therefore under-reported [26].

There are important limitations to this study. Because of the cross-sectional nature of this study, causative conclusions cannot be made. Although cotinine is a well-established biomarker for SHS exposure, the relatively short half-life of cotinine may underestimate cumulative SHS exposure, especially if the individual uses tobacco intermittently. This study categorized smoking status based on CDC definitions, and other categorization methods could offer different results. Furthermore, given the complicated and multifactorial nature of obesity, it is possible that obese patients have predisposing conditions related to hearing loss that were not explicitly measured. Medical history and loud noise exposure were self-reported and may be subject to recall bias. While sensorineural hearing loss was inferred by normal otoscopy and tympanometry, more rigorous evaluation would be needed to ascertain the type of hearing loss with full certainty. Results may not be generalizable to individuals outside of the U.S. Future research should also analyze potential differences between Never and Former smokers, which was infeasible in this study due to limitations related to sample size. Avenues for further research also include measuring other unmeasured covariates like head trauma, educational level, and other medical comorbidities that were unable to be included due to previously mentioned sample size constraints. The strengths of this study include providing further and more recent evidence for the association between SHS and hearing loss utilizing thresholds that align with the updated WHO guidelines. It analyzes hearing thresholds and hearing loss in a single data set with consistent analytical techniques, serving as a bridge between previous studies while including covariates not assessed prior. It also considers the better ear for more stringent analyses.

## Conclusion

This study builds on existing literature by supporting associations between SHS and hearing loss, finding several but not uniform effects. While the increases in hearing thresholds and odds of hearing loss are statistically significant, effect sizes appear to be relatively low and may not reach the threshold of clinical significance. Further research is needed to fully characterize the connection between obesity, SHS exposure, and hearing loss as well as determine the clinical significance of this relationship.
